# Application of Wavelet Entropy to Predict Atrial Fibrillation Progression from the Surface ECG

**DOI:** 10.1155/2012/245213

**Published:** 2012-09-26

**Authors:** Raúl Alcaraz, José J. Rieta

**Affiliations:** ^1^Innovation in Bioengineering Research Group, University of Castilla-La Mancha, Escuela Politécnica, Campus Universitario, 16071 Cuenca, Spain; ^2^Biomedical Synergy, Electronic Engineering Department, Universidad Politécnica de Valencia, 46730 Gandía, Spain

## Abstract

Atrial fibrillation (AF) is the most common supraventricular arrhythmia in clinical practice, thus, being the subject of intensive research both in medicine and engineering. Wavelet Entropy (WE) is a measure of the disorder degree of a specific phenomena in both time and frequency domains, allowing to reveal underlying dynamical processes out of sight for other methods. The present work introduces two different WE applications to the electrocardiogram (ECG) of patients in AF. The first application predicts the spontaneous termination of paroxysmal AF (PAF), whereas the second one deals with the electrical cardioversion (ECV) outcome in persistent AF patients. In both applications, WE was used with the objective of assessing the atrial fibrillatory (*f*) waves organization. Structural changes into the f waves reflect the atrial activity organization variation, and this fact can be used to predict AF progression. To this respect, results in the prediction of PAF termination regarding sensitivity, specificity, and accuracy were 95.38%, 91.67%, and 93.60%, respectively. On the other hand, for ECV outcome prediction, 85.24% sensitivity, 81.82% specificity, and 84.05% accuracy were obtained. These results turn WE as the highest single predictor of spontaneous PAF termination and ECV outcome, thus being a promising tool to characterize non-invasive AF signals.

## 1. Introduction

Atrial fibrillation (AF) is the most common cardiac arrhythmia, affecting almost 5% of the population older than 69 years of age and 8% of the population older than 80 years [1]. Although this arrhythmia itself does not represent a life-threatening condition, it predisposes to thrombus formation within the atria. As a consequence, AF increases mortality, stroke, and thromboembolism risks and reduces considerably the patients' quality of life [1]. Different AF subtypes can be specified depending on its usual evolution [2]. Paroxysmal atrial fibrillation (PAF) used to be the first one. In this stage, the arrhythmia terminates spontaneously without the need of medical intervention. The next stage is persistent AF, which requires pharmacological or electrical cardioversion (ECV) to allow its termination. Finally, the last stage is permanent AF, in which the termination is impossible or is not recommended, mainly, because of two reasons. On the one hand, the very low probability of AF reversion and, on the other hand, the high risks associated to the procedure [1].

Although the mechanism of AF is still unclear, it occurs when the electrical impulses in the atria degenerate from their usual organized pattern into a rapid chaotic pattern [1]. Thus, AF is associated with multiple meandering activation waves propagating randomly throughout the atria [3, 4]. The wavefronts fractionation, as they propagate, results in self-perpetuating independent wavelets, called reentries. The number of simultaneous reentries depends on refractory period, mass and conduction velocity of the atria [1]. This multiple wavelet hypothesis implies that the likelihood spontaneous AF termination is inversely related to the number of circulating wavelets in the atria [4]. Thus, self-sustained AF is associated with more circulating wavelets than nonsustained AF. In fact, previous works have suggested greater AF recurrence likelihood after ECV [5] and catheter ablation [6] in persistent AF patients with higher number of propagating reentries. 

 Even with this uncompleted understanding of AF mechanisms, several authors have demonstrated that a strict correlation between AF organization, defined as how repetitive is the AF signal pattern, and the number of wavefronts wandering the atrial tissue exists [3, 5]. Thereby, given that structural changes into surface fibrillatory (*f*) waves reflect the intraatrial activity organization variation [7], the aim of this work is to prospect the combination of Wavelet transform (WT) and entropy to predict organization-related events in AF from the surface ECG. In this sense, WT is a useful tool for the analysis of transients, aperiodicities, and other nonstationary signal features where subtle changes in signal morphology may be highlighted over different time-frequency scales [8]. Hence, given that entropy can be considered as a measure of the disorder degree of a signal [9], the application of entropy to the wavelet coefficients, referred to as Wavelet Entropy (WE) [10], would be a successful noninvasive estimator of *f* waves organization. 

 From a clinical point of view, the assessment of AF organization from the standard ECG is very interesting, since this recording can be easily and cheaply obtained and avoids the risks associated to invasive procedures [11]. On the other hand, given that about 18% of PAF patients degenerate into persistent AF in less than 4 years [12], the early prediction of AF maintenance is crucial. Thus, appropriate intervention may terminate the arrhythmia and prevent AF perpetuation. In contrast, the prediction of PAF termination could avoid unnecessary therapy, reduce the associated clinical costs, and improve the patient's quality of life. On the other hand, although ECV is a well-established strategy of AF therapy [13], arrhythmia recurrence is common during the first year after the procedure, even when the patients are under pharmacological therapy [13]. Therefore, the ECV success prediction could improve candidate selection for the procedure, thus reducing risks for the patient and costs for the healthcare provider. Overall, these two organization-dependent scenarios have been investigated, and WE diagnostic ability in the prediction of spontaneous PAF termination and ECV outcome in persistent AF patients have been analyzed.

## 2. Materials

 Two databases have been employed in the present study. First, a set of PAF recordings were analyzed to predict spontaneous termination of AF, and, secondly, a set of persistent AF recordings were studied to predict ECV outcome. The next subsections give additional details on these databases.

### 2.1. Paroxysmal AF Database

 Fifty Holter recordings of 30 seconds in length and two leads (II and V1) available in Physionet [14] were analyzed. The database included 26 nonterminating PAF episodes (group N), which were observed to continue in AF for, at least, one hour following the end of the excerpt, and 24 PAF episodes terminating immediately after the end of the extracted segment (group T). These signals were digitized at a sampling rate of 128 Hz and 16-bit resolution. Nonetheless, they were upsampled to 1024 Hz in order to allow better alignment for QRST complex subtraction, such as Bollmann et al. suggested [15]. This processing step is needed to extract the AA from the surface ECG, as will be next detailed in Section 3.1.

### 2.2. Persistent AF Database

 Sixty-three patients (20 men and 43 women, mean age 73.4 ± 9.0 years) with persistent AF lasting more than 30 days, referred to the Cardiology Department of the General University Hospital Consortium of Valencia (Spain) for ECV, were selected. They were followed during four weeks after ECV procedure. A standard 12-lead ECG was acquired for each patient during the whole procedure. The signals were digitized at a sampling rate of 1024 Hz and a resolution of 16 bits using a TEPA EKG Master USB recording system. A segment of 30 seconds in length preceding the cardioversion was extracted from each recording for the analysis. All the patients provided written informed consent for the study, which was approved by the human ethics committee of the hospital.

 After the ECV, 22 patients (34.93%) maintained NSR during the first month. On the contrary, in 31 patients (49.20%), NSR duration was lower than 1 month and the remaining 10 (15.78%) relapsed to AF immediately after ECV. These 41 patients constituted the group of AF recurrence. All patients were under drug treatment with amiodarone. The median arrhythmia duration was 10 months (range 1–47), and echocardiography demonstrated a mean left atrium diameter (LAD) of 45.82 ± 6.93 mm. Furthermore, 20.63% of the patients presented underlying heart disease. No significative differences were found in the aforementioned clinical parameters between the patients who maintained NSR and those others who relapsed to AF.

## 3. Methods

### 3.1. Data Preprocessing

 In both databases, lead *V*_1_ was chosen for the analysis because previous works have shown that AA is dominant in this lead [11]. This signal was preprocessed using forward/backward highpass filtering with 0.5 Hz cut-off frequency to remove baseline wander. Next, lowpass filtering with 70 Hz cut-off frequency was applied to reduce high frequency noise. Finally, notch filtering at 50 Hz was applied to remove powerline interference [16]. In addition, a reliable analysis of the AA from the surface ECG requires that ventricular activity has been cancelled previously [15]. Although a variety of different techniques exist for this purpose, an adaptive singular value QRST cancellation template was applied [17].

### 3.2. Wavelet Transform

 From a mathematical perspective, the *wavelet* is a smooth and quickly vanishing oscillating function with good localization in both time and frequency. A *wavelet family *Ψ_*a*,*b*_(*t*) is the set of elementary functions generated by dilations and translations of a unique admissible *mother wavelet *Ψ(*t*) [8], that is:

(1) $$\begin{array}{c} \Psi_{a,b}\left(t\right)={\left|a\right|}^{-(1/2)}\Psi \left(\frac{t-b}{a}\right), \end{array}$$

where *a*, *b* ∈ *ℜ*, *a* ≠ 0 are the scale and translation parameters, respectively, and *t* is the time. As *a* increases, the wavelet becomes narrower. Thus, one has a unique analytic pattern and its replications at different scales and with variable time localization. 

 The Discrete Wavelet Transform (DWT) is the sampled version of the Continuous Wavelet Transform (CWT) in a dyadic grid employing orthonormal wavelet basis functions [8]. Hence, the parameters *a* and *b* are sampled using a logarithmic discretization of the *a* scale (*a* = 2^*m*^), and this, in turn, is linked to the steps size taken between the *b* locations. To link *b* to *a*, each location *b*, which is proportional to the *a* scale, is moved in discrete steps (*b* = *n* · 2^*m*^). Thus, the discretized mother wavelet is

(2) $$\begin{array}{c} \Psi_{m,n}\left(k\right)=2^{-(m/2)}\Psi \left(2^{-m}k-n\right), \end{array}$$

with *m* and *n* being the new scale and translation discrete parameters, respectively, and *k* the discrete time instant. Hence, the wavelet decomposition of the AA signal, *x*_AA_(*k*), can be defined as its correlation with the chosen wavelet family Ψ_*m*,*n*_(*k*) for each *m* and *n*, that is:

(3) $$\begin{array}{c} C_{m}\left(n\right)=\sum_{k}x_{\text{AA}}\left(k\right)\cdot \Psi_{m,n}\left(k\right). \end{array}$$

The decomposition results in wavelet coefficients *C*, which depend on scale and position. In fact, a vector of wavelet coefficients **C**_*m*_ is obtained for each analyzed discrete scale *m*. The information stored in the wavelet coefficients vectors is not repeated elsewhere and allows the complete regeneration of the original signal without redundancy, because the used discretization of the mother wavelet employs orthonormal basis functions [8].

### 3.3. Wavelet Entropy

 The entropy of a random variable reflects the degree of disorder that the variable possesses. The more uncertain the variable is, the greater its entropy [9]. Entropy, *H*, for a discrete random variable *X* is defined as

(4) $$\begin{array}{c} H\left(X\right)=-\sum_{i}P\left(X=a_{i}\right)\log P\left(X=a_{i}\right), \end{array}$$

where *a*_*i*_ are the possible values of *X*. The conventional definition of entropy is described in terms of the temporal distribution of signal energy in a given time window. The distribution of energy in a specified number of data values intervals is described in terms of the probabilities in signal space {*p*_*i*_}, where *p*_*i*_ is the probability that *X* = *a*_*i*_ [9]. 

 In an orthonormal basis the concept of energy is linked with the usual notions derived from the Fourier theory [10]. Then, the relative energy of the wavelet coefficients at each scale *m* can be expressed as

(5) $$\begin{array}{c} E_{m}=\frac{\sum_{i}{\left|C_{m}\left(i\right)\right|}^{2}}{\sum_{m=1}^{N}\sum_{i}{\left|C_{m}\left(i\right)\right|}^{2}}, \end{array}$$

with *N* being the number of wavelet decomposition levels. Clearly ∑_*m*=1_^*N*^ *E*_*m*_ = 1 and the distribution {*E*_*m*_} can be considered as a time-scale density, which is a suitable tool for detecting and characterizing specific phenomena in time and frequency domains [10]. Therefore, WE can be defined as

(6) $$\begin{array}{c} \text{WE}=-\sum_{n=1}^{N}E_{m}\log\left(E_{m}\right), \end{array}$$

being a measure of the degree of order/disorder of the signal, so it can provide useful information about the underlying dynamical process associated with the signal [10]. To this respect, for a very organized signal, such as a periodic monofrequency signal, WE provides a very low value near zero, given that its wavelet decomposition shows a relative wavelet energy near one for the level containing the representative frequency of the signal and a very limited relative energy for the remaining wavelet levels. In contrast, a very disorganized signal, such as those generated by a totally random process, has a wavelet representation with significant contributions from all the frequency bands, thus providing a high WE value near its maximum. 

 The application of WE to the AA signal requires the appropriate selection of the number of decomposition levels and a mother wavelet function. In this sense, a seven-level decomposition was chosen, given that the seventh scale resulted in a good match to the AA frequency bands of interest [18], that is, 4–8 Hz, and it has provided successful outcomes in previous works dealing with AF [19, 20]. Regarding the wavelet family selection, there are no established rules for the choice of wavelet functions. A cautious and still exploratory approach is to test different wavelet families and then to compare their efficiency in the specific problem [21]. Unfortunately, on each electrocardiographic application where the WT has been used, a different wavelet family was chosen [22]. In this study, several orthogonal wavelet families were tested, because only in an orthogonal basis any signal can be uniquely decomposed, and the decomposition can be inverted without losing information [8].

### 3.4. Statistical Analysis

In order to evaluate WE diagnostic ability from each considered AF scenario, a stratified 2-fold cross-validation was used. Thus, the database was first partitioned into 2 equally sized folds. Subsequently, 2 iterations of training and validation were performed, such that with each iteration a fold of the data was held out for validation while the other was used for learning. From each learning set, optimum WE threshold was computed making use of a receiver operating characteristic (ROC) curve. It was created by plotting the fraction of true positives out of positives (sensitivity) versus the fraction of false positives out of the negatives (1-specificity) at various threshold settings. The threshold value providing the highest percentage of patients correctly classified, that is, accuracy, was selected as optimum WE threshold. This value was thereafter used to compute sensibility, specificity, and accuracy from the corresponding validation set. It is worth noting that data was stratified prior to being split into 2 folds. Stratification is the process of rearranging the data as to ensure each fold is a good representative of the whole. 

 On the other hand, for both learning and validation sets, significant differences between terminating and nonterminating PAF episodes and between patients who resulted in NSR and relapsed to AF were evaluated making use of Student's *t*-test. All the groups had a normal and homoscedastic distribution as the Shapiro-Wilk and Levene tests proved, respectively. A two-tailed value of statistical significant *P* < 0.05 was considered statistically significant. Finally, note that all the ECG preprocessing, WE, and statistical tests were computed under MATLAB 7.12 (The MathWorks Inc., Natick, Massachusetts, USA) on a personal computer.

## 4. Results

 As the number of episodes considered for each database was not notably large, a stratified 2-fold cross-validation was run five times for each considered AF scenario, 10 learning and 10 validation sets thus being analyzed. Therefore, sensitivity, specificity, and accuracy values that will be presented in the next subsections were averaged for the corresponding 10 folds. It has to be noted that, for the prediction of spontaneous PAF termination, sensitivity was considered as the proportion of nonterminating PAF episodes correctly discerned, whereas specificity represented the percentage of terminating episodes properly identified. Similarly, for the ECV outcome analysis, sensitivity was the proportion of patients relapsing to AF appropriately classified, and specificity was the percentage of patients resulting in NSR accurately predicted after ECV.

### 4.1. Learning Sets

 All the different functions from Haar, Daubechies, Coiflet, Biorthogonal, Reverse Biorthogonal, and Symlet wavelet families were tested from all the considered learning sets for both paroxysmal and persistent AF databases. All the functions coming from the same wavelet family provided similar statistical significance values, and the same sensitivity, specificity, and accuracy were noticed for each analyzed learning set. Thus, only the function that presented the lower *P* value is included for each wavelet family in Tables 1 and 2, which present averaged WE, sensitivity, specificity, and accuracy values for PAF termination and ECV result predictions, respectively. As can also be appreciated in these tables, all the wavelet families reached the same discriminant ability for each analyzed scenario. Moreover, the same patients were incorrectly classified by all the families for every learning set. Consequently, any wavelet family could be used indistinctly. Nonetheless, both for PAF termination and ECV result predictions, the highest statistical differences between patient groups were noticed for the biorthogonal wavelet family of order (4,4), such as in previous works [19, 20]. Thus, considering that only one wavelet function is required to compute WE, the aforementioned biorthogonal family was selected.

### 4.2. Validation Sets

**Figure 1:**
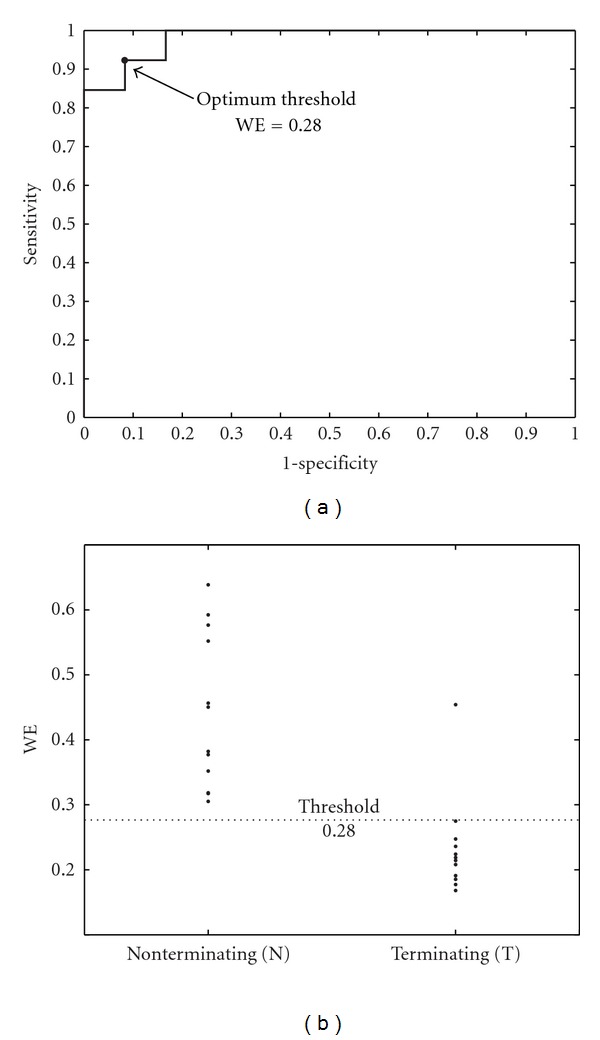
(a) ROC curve constructed from a specific learning set of the PAF patients. The WE value providing the highest accuracy was selected as optimum threshold, which has been marked with symbol •. (b) Classification into terminating and nonterminating PAF episodes for the corresponding validation set making use of the WE threshold obtained from the previous ROC curve.

**Figure 2:**
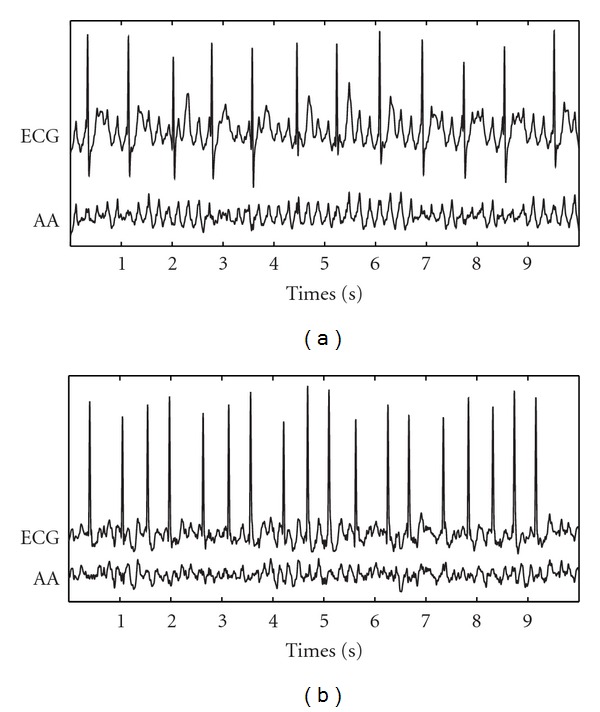
Typical ECG interval together with its extracted AA signal for (a) a terminating (WE = 0.189) and (b) other nonterminating (WE = 0.452) PAF episodes.

 For PAF termination prediction, ROC curves from the learning sets provided optimum WE discrimination thresholds between 0.26 and 0.33. With these thresholds, sensitivity, specificity, and accuracy mean values for the validation sets were 95.38%, 91.67%, and 93.60%, respectively. As for learning sets, nonterminating PAF episodes presented higher WE values (0.452 ± 0.018, in average) than the terminating ones (0.231 ± 0.013, in average), both PAF groups being statistically distinguishable for the 10 analyzed cases (*P* < 0.001). As an example, Figure 1 shows the ROC curve computed from a specific learning set and classification into terminating and nonterminating PAF episodes for the corresponding validation set. Moreover, with the aim of illustrating the *f* waves disorder degree quantified by WE, Figure 2 presents a 10-second-length ECG interval together with its extracted AA signal for a typical terminating PAF episode and other nonterminating ones.

**Figure 3:**
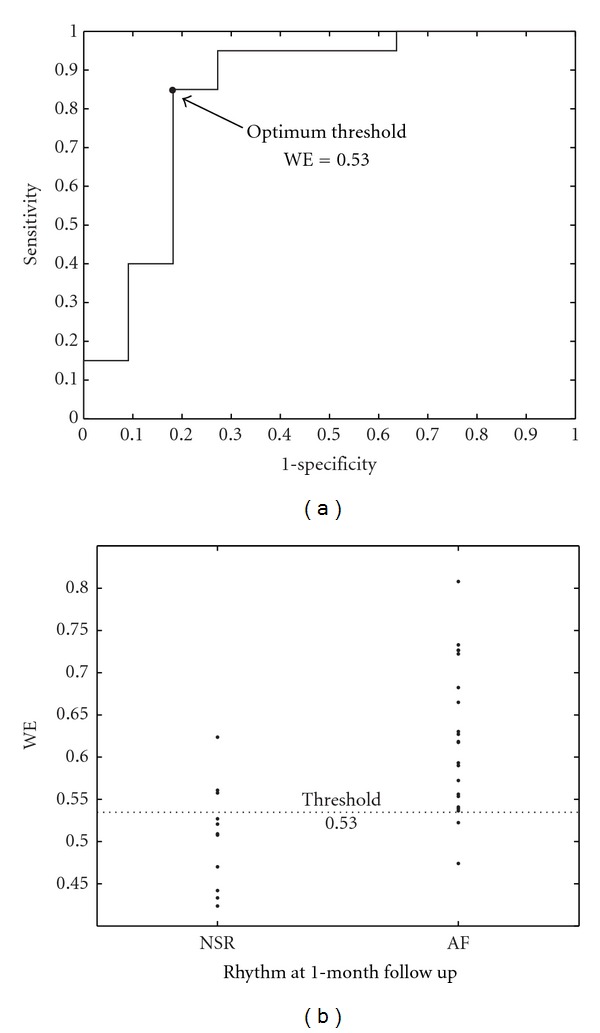
(a) ROC curve constructed from a specific learning set of the persistent AF patients. The WE value providing the highest accuracy was selected as optimum threshold, which has been marked with symbol •. (b) Classification into ECV patients resulting in NSR and relapsing to AF for the corresponding validation set making use of the WE threshold obtained from previous ROC curve.

**Figure 4:**
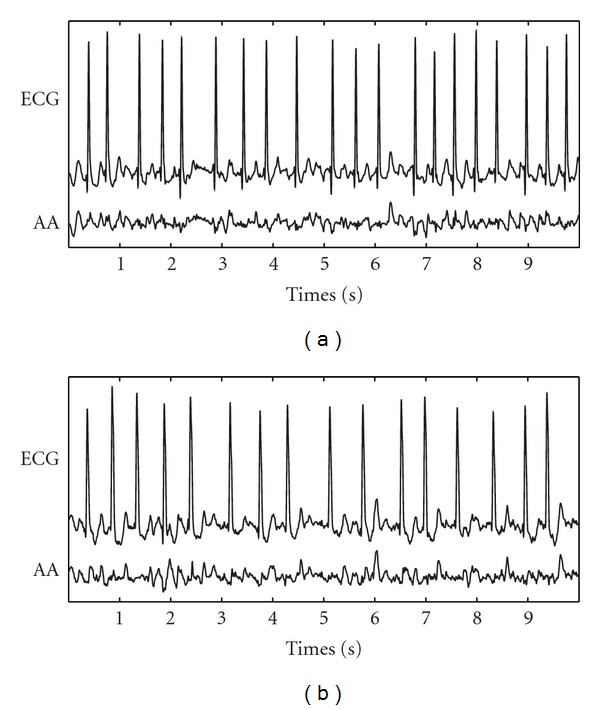
Typical ECG interval together with its extracted AA signal for (a) a patient who maintained NSR (WE = 0.511) and (b) others relapsing to AF (WE = 0.633) during the first month after cardioversion.

Regarding ECV outcome prediction, ROC curves from the learning sets provided optimum thresholds between 0.51 and 0.57, with which sensitivity, specificity, and accuracy values, averaged for the corresponding validation sets, of 85.24%, 81.82%, and 84.05% were noticed. To this respect, Figure 3 displays ROC curve obtained from a specific learning set and the performance classification for the corresponding validation set. As in this figure, the patients relapsing to AF presented higher WE values (0.635 ± 0.026, in average) than those resulting in NSR after one month (0.508 ± 0.022, in average), considering all the validation sets. In addition, statistical significance was lower than 0.001 for all the studies cases. Finally, Figure 4 displays a 10-second-length ECG signal together with its extracted AA signal for a typical patient maintaining NSR and other relapsing to AF during the first month after cardioversion. Note that more irregular *f* waves than for paroxysmal AF patients (Figure 2) can be appreciated.

## 5. Discussion

 To date, several single predictors of spontaneous PAF termination and ECV result have been proposed from the surface ECG analysis in time and frequency domains. From the time point of view, the application of a nonlinear regularity index as sample entropy (SampEn) to the main atrial wave (MAW), that is, the fundamental waveform of the AA signal, to estimate noninvasively AF organization has provided a diagnostic ability of 90% in PAF termination prediction [23] and approximately 80% in ECV outcome prediction [24]. The amplitude of *f* waves has also proved its ability to predict ECV outcome with an accuracy near 80% [25]. Regarding the frequency domain, most works have analyzed the dominant atrial frequency (DAF) of the AA signal, providing a diagnostic ability in PAF termination prediction between 86% and 90%, depending on the used methodology [26, 27]. In contrast, conflicting outcomes have been obtained when the DAF was applied to ECV outcome prediction [28]. Some authors [29] suggested that the confounding effect of antiarrhythmic drug therapy could explain the differences among these results; however, this aspect still remains unclear [28]. Anyway, outcomes reported in the present work outperform all the single predictors published in previous works, given that the discriminant ability of WE was 94% in the prediction of PAF termination and 84% in the prediction of ECV outcome, respectively. A possible justification for this finding could be that wavelet analysis can capture AA signal characteristics from both time and frequency domains with high precision [8]. In fact, WE also surpassed the results provided by single predictors based on the application of different time-frequency transforms to both analyzed AF scenarios [27, 30]. 

 In the literature, single parameter combinations and advanced classification tools have also been proposed to predict AF events from the surface ECG. Thus, regarding PAF termination prediction, DAF has been combined with other parameters to improve its diagnostic accuracy. To this respect, Petrutiu et al. [31] studied the AA peak frequency power evolution within the last two seconds before spontaneous PAF termination, reaching thus an accuracy of 93.33%. The same classification result was reported by combining SampEn and WT [19]. In that work, SampEn was applied to the wavelet coefficient vector containing the DAF and its reconstruction to the time domain, providing two different and independent classifications, which were combined as a function of the DAF. A slightly higher result (accuracy of 96.67%) was reached by Sun and Wang [32] making use of a multilayer perceptron neural network to combine 11 features extracted from the ECG recurrence plot quantification. With regard to ECV outcome prediction, Watson et al. [33] examined a variety of wavelet transform-based statistical markers, which obtained a sensitivity of 88% and specificity of 100% by means of a nonparametric classification system. Žohar et al. [34] developed a nondeterministic model with several parameters as inputs for predicting NSR maintenance after ECV, providing a diagnostic accuracy of 84%. As for PAF termination prediction, the AA signal organization estimation both in time and wavelet domains through SampEn provided a sensitivity of 95% and specificity of 93% [20]. However, remark that in this study only patients reverting back to NSR in only one attempt of ECV were analyzed and, therefore, with AA signals notably organized [24]. Finally, recent works have reported that the combination of *f* waves amplitude with the DAF [25] or SampEn [24] computed from the MAW reached a discriminant ability of 86 and 90%, respectively. 

 As a consequence, it can be asserted that only complex combinations of single predictors can improve the WE classification result. Thereby, WE can be considered as a promising single estimator of PAF termination and ECV outcome, with the additional advantage of a simpler implementation to work in real-time. To this respect, DWT can be computed efficiently with a pyramid filter bank algorithm [8], thus allowing its implementation in real-time [35]. In contrast, the classical algorithm proposed in the literature for SampEn computation requires a high execution time, which is not fast enough for online applications [36]. Although faster alternatives for SampEn computation have been recently proposed [36], their ability to be implemented in a real-time environment has not been proved yet. In addition, accuracy of these new algorithms has not been validated by comparison with the classical SampEn definition. On the other hand, it has to be remarked that the complex combination of multiple parameters or the use of advanced classification techniques, such as in [32] or in [33], turns difficult a direct and clear clinical interpretation of the results. In this sense, possible clinical meaning of each parameter is blurred within the classification approach. Nonetheless, remark that a simple combination of WE with other parameters could be interesting to improve its diagnostic ability. Successful predictors previously presented, such as the DAF or the *f* waves amplitude, together with new features computed from the wavelet coefficients could be considered for this purpose. However, further studies would be required in this line, given that only a relevant improvement can be reached through the use of metrics providing complementary information to WE. 

On the other hand, terminating PAF episodes presented lower WE values than nonterminating ones, see Figure 1, suggesting more structured and regular *f* waves in patients with immediate spontaneous reversion to NSR. This finding is in agreement with the decrease in the number of reentries prior to NSR restoration observed in previous invasive studies, where AF termination was achieved by using different therapies [3, 6]. This decrease in the number of reentries produces simpler wavefronts into the atrial tissue, and irregular *f* waves evolve to regular *P* waves [11]. With respect to ECV, patients who relapsed to AF presented higher WE values than those who remained in NSR; see Figure 3. This finding, suggesting more organized AA signals in effective cardioversions one month after the procedure, agrees with observations obtained from previous works, such as (i) the higher the AA organization, the higher the success rate in AF cardioversion [5], (ii) the higher the AA organization, the lower the energy required for successful cardioversion [37], and (iii) PAF requires less energy for cardioversion than persistent AF [38]. These observations highlight the fact that, when a higher number of reentries are wandering throughout the atrial tissue, a lower probability of successful ECV is obtained. One possible explanation could be that a low degree of AA organization might result in an increased mass of atrial myocardium that is not fully excitable [37]. Furthermore, other interesting observation is that all the PAF patients showed lower WE values than those with persistent AF undergoing ECV, suggesting more irregular and non-structured*f* waves in persistent than in paroxysmal AF. This finding agrees with the results reported through invasive studies in humans [39] and dogs [40], which showed that persistent AF presents a higher degree of disorganized activity in the atria than PAF. 

 From a noninvasive point of view, results obtained with WE also were in agreement with those provided by previous works in which *f* waves organization was estimated. To this respect, the independent application of SampEn to the MAW and to the wavelet coefficient vector containing the DAF showed more regular *f* waves for terminating PAF episodes than for nonterminating ones [19] and for patients who maintained NSR than for those relapsing to AF after ECV [20]. However, the diagnostic accuracy of these two independent estimates of *f* waves regularity was below WE, being around 90% and 80% for PAF termination and ECV outcome prediction, respectively. Nonetheless, it is noteworthy that the WE philosophy is completely different to the idea previously developed, in which only the wavelet coefficients vector regularity for a scale was considered [19, 20]. In contrast, the relative energy carried by the wavelet coefficients vector in all the decomposition levels is considered for the computation of WE. 

 Furthermore, it has to be remarked that, for a specific wavelet scale, its corresponding vector contains the correlation coefficients between the scaled mother wavelet and the consecutive and nonoverlapping signal segments. Hence, it is plausible to consider that the relative energy of each scale remains without notable alterations, although the mother wavelet would change. In this respect, results provided by all the tested wavelet families can be considered as coherent. Nonetheless, as in previous works [19, 20], the biorthogonal wavelet family provided the highest statistical differences between groups, which could be due to the lowest phase distortion produced by the filters of this family [8]. 

 Finally, some limitations merit consideration. First, the analysis was developed with a limited number of patients. A larger sample, allowing a more rigorous statistical study, is required to provide improved confidence in the robustness of the developed approach. To this respect, wider databases containing nonterminating and spontaneously terminating PAF episodes after different time epochs (ten minutes, half an hour, an hour, ten hours, etc.) and patients who resulted in NSR and relapsed to AF after 3, 6, and 12 months following ECV would be needed. Second, the persistent AF database only included suitable ECV patients following the standard clinical criteria; therefore, it is unknown how WE will behave in patients with adverse clinical predictors, like atrial dilatation, and so forth, which, by default, are excluded from ECV procedures. Finally, only lead *V*_1_ was analyzed rejecting the possible information contained in the remaining leads. However, for this type of studies, lead *V*_1_ seems to be the most suitable lead. In this respect, significant correlations between the information in lead *V*_1_, such as the DAF or SampEn, and invasive atrial electrograms have been reported [7].

## 6. Conclusions

The present work has demonstrated that WE is able to evaluate *f* waves organization from the surface ECG. WE has proved to be the highest single predictor of spontaneous PAF termination and ECV outcome published to date. Nonetheless, although complex and advanced combinations of other parameters measured from the ECG can improve its diagnostic ability, the application of WE has two interesting advantages: a clear clinical interpretation of the results and the possibility of working in a real-time environment. This parameter may lead towards the development of improved therapeutic interventions for the treatment of paroxysmal and persistent AF, since useless procedures could be avoided and the consequent risk for AF patients could be minimized.

## Figures and Tables

**Table 1:** Mean and standard deviation of WE values for nonterminating and terminating PAF groups, statistical significance (*P* value), sensitivity, specificity, and accuracy for each studied wavelet family. These values were computed and averaged for the 10 analyzed learning sets.

Wavelet family (Order)	Group N	Group T	*P* value	Sensitivity	Specificity	Accuracy
Haar	0.231 ± 0.012	0.441 ± 0.018	<0.001	96.15%	93.75%	95%
Daubechies (*5*)	0.252 ± 0.021	0.520 ± 0.029	<0.001	96.15%	93.75%	95%
Coiflet (*3*)	0.211 ± 0.019	0.487 ± 0.014	<0.001	96.15%	93.75%	95%
Biorthogonal (4.4)	0.231 ± 0.022	0.452 ± 0.029	<0.001	96.15%	93.75%	95%
Reverse biorthogonal (4.4)	0.290 ± 0.041	0.522 ± 0.035	<0.001	96.15%	93.75%	95%
Symlets (*5*)	0.250 ± 0.023	0.524 ± 0.028	<0.001	96.15%	93.75%	95%

**Table 2:** Mean and standard deviation of WE values for patients relapsing to AF and maintaining NSR during the first month following cardioversion, statistical significance (*P* value), sensitivity, specificity, and accuracy for each studied wavelet family. These values were computed and averaged for the 10 analyzed learning sets.

Wavelet family (Order)	ECVs relapsing to AF	ECVs maintaining NSR	*P* value	Sensitivity	Specificity	Accuracy
Haar	0.627 ± 0.021	0.512 ± 0.025	<0.001	87.74%	79.09%	84.75%
Daubechies (*5*)	0.642 ± 0.029	0.521 ± 0.034	<0.001	87.74%	79.09%	84.75%
Coiflet (*3*)	0.612 ± 0.013	0.502 ± 0.020	<0.001	87.74%	79.09%	84.75%
Biorthogonal (4.4)	0.625 ± 0.023	0.508 ± 0.018	<0.001	87.74%	79.09%	84.75%
Reverse Biorthogonal (4.4)	0.651 ± 0.044	0.549 ± 0.038	<0.001	87.74%	79.09%	84.75%
Symlets (*5*)	0.630 ± 0.031	0.528 ± 0.033	<0.001	87.74%	79.09%	84.75%
